# Supplementary material for: Prevalence and outcomes of patients developing heparin-induced thrombocytopenia during extracorporeal membrane oxygenation
Source: PLoS One. 2022 Aug 8;17(8):e0272577. doi: 10.1371/journal.pone.0272577 (PMC9359525; doi:10.1371/journal.pone.0272577)
Supplement: S1 File — (PDF) [file pone.0272577.s001.pdf]

S1 File. Supplementary materials

4 T-Score for diagnosis of HIT

	2 points	1 point	0 points
**Thrombocytopenia**	fall in platelet count is >50% of the previous value, AND the lowest count (nadir) is 20–100 × 10^9/liter	fall is 30–50% or the nadir is 10–19 × 10^9/liter	fall is less than 30% or the nadir is <10 × 10^9/liter
**Timing**	fall is between days 5–10 after commencement of treatment<br>or on day 0-1 after heparin exposition during the last 30 days	fall is after day 10 or on day 0-1 after heparin exposition during the last 30 - 100 days	fall is early (before 5th day) but there has been no previous heparin exposure
**Thrombosis**	new proven thrombosis, skin necrosis, or systemic reaction to heparin administration	progressive or recurrent thrombosis, silent thrombosis or thrombosis suspicion, red skin lesions to heparin injection	no symptoms
**Alternative cause for thrombocytopenia possible**	no other cause	possible alternative cause	definite alternative cause

Score 0–3: HIT unlikely.
Score 4–5: intermediate HIT probability
Score 6–8: very high HIT probability

Keeling D, Davidson S, Watson H (May 2006). "The management of heparin-induced thrombocytopenia". Br. J. Haematol. ***133*** *(3): 259–69. doi:10.1111/j.1365-2141.2006.06018.x. PMID 16643427.*
Warkentin TE, Heddle NM (March 2003). "Laboratory diagnosis of immune heparin-induced thrombocytopenia". Curr Hematol Rep. ***2*** *(2): 148–57. PMID 12901146.*

Used ECMO systems:

PLS-system and Cardiohelp-system with heparin based Bioline-coating Gettinge / Maquet, Rastatt, Germany.
Hilite7000LT oxygenator + DP3 pump with heparin based Rheoparin-coating, Medos Medizintechnik, Stolberg, Germany; iLA-activve-system with heparin based Rheoparin-coating, Fresenius Medical Care / Xenios / Novalung, Heilbronn, Germany.
ECC.05 system with Phosphorylcholin-coating, Livanova / Sorin Group, Modena, Italy.

Laboratory tests:

HIT antibodies:

The HIT-antibody detection was done with an ELISA (PF4-IgG, Immucor GTI) until 2013, thereafter a CLIA (Chemiluminescene Immunoassay) (HemosIL AcuStar HIT-IgG, IL) was used.

Heparin induced Platelet Aggregation Test (HIPA):

The HIPA test is done externally at the Department of Transfusion medicine University Medical Center Greifswald lead by Prof. Dr. A. Greinacher. Details were published earlier. Thromb Res 2017. doi:10.1016/j.thromres.2017.08.008.

D-Dimer-Test:

Until 03/2008: DDimer PLUS, Fa. Siemens, unit: µg/l, which had to be changed because reagent was no longer produced.
From 03/2008 up to now: INNOVANCE D-Dimer, Fa. Siemens, unit: mg/l.
