# Supplementary material for: Prevalence and outcomes of patients developing heparin-induced thrombocytopenia during extracorporeal membrane oxygenation
Source: PLoS One. 2022 Aug 8;17(8):e0272577. doi: 10.1371/journal.pone.0272577 (PMC9359525; doi:10.1371/journal.pone.0272577)
Supplement: S1 Table — (PDF) [file pone.0272577.s002.pdf]

S1 Table. Characteristics of patients with confirmed heparin-induced thrombocytopenia

Patient	Age, y	Sex	SOFA	LIS	Time to HIT, days	Days on ECMO	Survivor	ECMO mode	Bleeding	Technical problem	Thrombo-embolism	Platelet count / ECMO begin[illegible] /nadir
1	35	F	12	3	9	24	No	VV	No	Yes	No	178/106
2	72	M	9	3	12	7	Yes	VA	No	No	No	130/15
3	64	M	9	0	12	17	Yes	VV	Yes	No	Yes	354/88
4	51	M	6	4	7	8	No	VA	No	No	Yes	260/115
5	45	M	13	4	9	26	Yes	VV	No	Yes	No	222/25
6	53	F	11	4	7	10	Yes	VV	No	No	Yes	369/49
7	65	M	13	4	12	27	No	VA	Yes	No	Yes	133/42
8	58	M	9	3	5	5	Yes	VV	No	No	Yes	286/34
9	61	F	-	2	5	14	Yes	VV	Yes	Yes	No	136/42
10	74	M	14	4	4	23	No	VV	No	No	Yes	200/58
11	73	M	-	3	4	9	Yes	VV	No	Yes	Yes	328/78
12	50	M	15	4	12	43	No	VV	Yes	No	Yes	241/36
13	50	M	18	3	1	11	Yes	VV	Yes	No	Yes	56/49
14	64	M	15	4	9	10	Yes	VV	Yes	Yes	No	398/70
15	28	F	1	0	5	36	No	VV	No	Yes	No	185/52
16	41	M	-	2	8	10	Yes	VV	No	No	Yes	71/11
Ø	55.5	F 4 (25%)<br>M 12 (75%)	12	3	7.5	12.5	Survivor 10 (62.5%)<br>Nonsurvivor 6 (37.5%)	VA 3 (18.8%)<br>VV 13 (81.2%)	Yes 6 (37.5%)<br>No 10 (62.5%)	Yes 6 (38%)<br>No 10 (63%)	Yes 10 (63%)<br>No 6 (38%)	221/49

∅: median or sum; F: female; M: male; SOFA: sequential organ failure assessment; LIS: lung injury score; HIT: heparin-induced thrombocytopenia; ECMO: extracorporeal membrane oxygenation; VV: veno-venous; VA: veno-arterial; Time to HIT: Time between ECMO initiation and change of anticoagulation to argatroban.
