# Supplementary material for: Prevalence and outcomes of patients developing heparin-induced thrombocytopenia during extracorporeal membrane oxygenation
Source: PLoS One. 2022 Aug 8;17(8):e0272577. doi: 10.1371/journal.pone.0272577 (PMC9359525; doi:10.1371/journal.pone.0272577)
Supplement: S2 Table — (PDF) [file pone.0272577.s003.pdf]

S2 Table. Localisation of Thrombotic Events

	**HIT-confirmed**	**HIT-suspicion**	**HIT-excluded**	**ECMO-control**	**Total**	**P-value**
Arterial events	n = 3	n = 0	n = 6	n = 30	n = 39	0.014
Intracardiac thrombus formation n (%)	0 (0%)	-	1 (17%)	2 (7%)	3 (8%)	
Cerebral ischemia n (%)	1 (33%)	-	0 (0%)	0 (0%)	1 (3%)	
Thrombus in cannulated artery n (%)	1 (33%)	-	2 (33%)	5 (17%)	8 (21%)	
Ischemia of cannulated leg with intervention n (%)	0 (0%)	-	1 (17%)	20 (69%)	21 (55%)	
Other artery n (%)	1 (33%)	-	2 (33%)	2 (7%)	5 (13%)	
Venous events	n = 9	n = 4	n = 23	n = 113	n = 149	<0.001
Cannulated vessel n (%)	5 (56%)	1 (25%)	12 (52%)	93 (82%)	111 (75%)	
Vena Cava inferior n (%)	0 (0%)	1 (25%)	5 (22%)	5 (4%)	11 (7%)	
Pulmonary embolism n (%)	3 (33%)	1 (25%)	5 (22%)	14 (12%)	23 (15%)	
Other veins n (%)	1 (11%)	1 (25%)	1 (4%)	1 (1%)	4 (3%)	

Summary of all thrombotic events. More than one thrombotic event was observed in four patients of the group HIT-confirmed, in three patients of the group HIT-suspicion, in nine patients of the group HIT-excluded and in thirty patients of the ECMO control group but only the most important venous thrombotic event is reported. p-value for intergroup differences of either arterial or venous thrombotic events.

HIT: heparin-induced thrombocytopenia; ECMO: extracorporeal membrane oxygenation; other artery: radial artery or femoral artery; other veins: posterior tibial vein, fibular vein.
