# Supplementary material for: Prevalence and outcomes of patients developing heparin-induced thrombocytopenia during extracorporeal membrane oxygenation
Source: PLoS One. 2022 Aug 8;17(8):e0272577. doi: 10.1371/journal.pone.0272577 (PMC9359525; doi:10.1371/journal.pone.0272577)
Supplement: S3 Table — (PDF) [file pone.0272577.s004.pdf]

S3 Table. Technical problems

	HIT-confirmed	HIT-suspicion	HIT-excluded	ECMO-control	Total	P-value
Number of technical events	n = 6	n = 1	n = 24	n = 51	n = 82	0.103
Pump-head thrombosis, n (%)	2 (33)	0 (0%)	7 (29)	14 (28)	23 (28)	
Oxygenator/System thrombosis, n (%)	2 (33)	0 (0%)	8 (33)	9 (17)	19 (23)	
Cannula thrombosis, n (%)	0 (0%)	0 (0%)	2 (8)	3 (6)	5 (6)	
Plasma leak of Oxygenator, n (%)	0 (0%)	1 (100)	0 (0%)	5 (10)	6 (7)	
Impaired gas transfer, n (%)	2 (33)	0 (0%)	7 (29)	20 (39)	29 (35)	

Summary of all technical problems. More than one technical problem was observed in one patient with confirmed HIT, in ten patients in the HIT excluded group and in thirty patients in the ECMO control group but only the most important technical problem is reported. p-value for intergroup differences of technical problems. HIT: heparin-induced thrombocytopenia; ECMO: extracorporeal membrane oxygenation.
