# Supplementary material for: Prevalence and outcomes of patients developing heparin-induced thrombocytopenia during extracorporeal membrane oxygenation
Source: PLoS One. 2022 Aug 8;17(8):e0272577. doi: 10.1371/journal.pone.0272577 (PMC9359525; doi:10.1371/journal.pone.0272577)
Supplement: S4 Table — (PDF) [file pone.0272577.s005.pdf]

S4 Table. Bleeding Events

	HIT-confirmed	HIT-suspicion	HIT-excluded	ECMO-control	Total	P-value
Number of bleeding events	n = 6	n = 3	n = 22	n = 54	n = 85	0.694
Cerebral, n (%)	1 (17)	1 (33)	5 (24)	10 (18)	17 (20)	
Pulmonary, n (%)	0 (0)	1 (33)	3 (14)	3 (7)	7 (8)	
Gastrointestinal, n (%)	2 (33)	0 (0)	5 (24)	5 (9)	12 (14)	
Wound, n (%)	1 (17)	0 (0)	0 (0)	3 (5)	4 (5)	
Diffuse, n (%)	1 (17)	0 (0)	1 (5)	10 (18)	12 (14)	
Retroperitoneal, n (%)	0 (0)	1 (33)	2 (10)	6 (11)	9 (11)	
Insertion site, n (%)	0 (0)	0 (0)	3 (14)	10 (18)	13 (15)	
Oral, n (%)	1 (17)	0 (0)	1 (5)	3 (5)	5 (6)	
Others, n (%)	0 (0)	0 (0)	2 (5)	4 (9)	6 (7)	

Summary of all bleeding events. More than one bleeding events was observed in two patients with confirmed HIT, in three patients in the HIT suspicion group, in four patients in the HIT excluded group and in nine patients in the ECMO control group but only the most important bleeding event is reported. p-value for intergroup differences of bleeding events. HIT: heparin-induced thrombocytopenia; ECMO: extracorporeal membrane oxygenation; p-value: 0,694, others = intercostal arteries, lack puncture, thoracic.
