# Supplementary material for: Prevalence and outcomes of patients developing heparin-induced thrombocytopenia during extracorporeal membrane oxygenation
Source: PLoS One. 2022 Aug 8;17(8):e0272577. doi: 10.1371/journal.pone.0272577 (PMC9359525; doi:10.1371/journal.pone.0272577)
Supplement: S5 Table — (PDF) [file pone.0272577.s006.pdf]

S5 Table. Consumption of blood products on extracorporeal membrane oxygenation

	HIT-confirmed (n=16)	HIT-suspicion (n=10)	HIT-excluded (n=55)	ECMO-control (n=426)	P-value between groups
Packed red blood cells	2.5 (0.3-6.0)	4.5 (2-13)	5.0 (1-13)	1.5 (0-5)	<0.001[1.2]
Erythrocyte concentrate per day	0.2 (0.0-0.3)	0.3 (0.1-0.8)	0.1 (0.3-0.6)	0.1 (0.0-0.5)	0.049
Fresh frozen plasma	0.0 (0.0-0.8)	0.0 (0.0-1.5)	0.0 (0.0-4.0)	0.0 (0.0-4.0)	0.827
Fresh frozen plasma per day	0.0 (0.0-0.03)	0.0 (0.0-0.06)	0.0 (0.0-0.19)	0.0 (0.0-0.3)	0.718
Thrombocyte concentrate	0.0 (0.0-0.8)	0.0 (0.0-0.0)	0.0 (0.0-1.0)	0.0 (0.0-0.0)	0.053
Thrombocyte concentrate per day	0.0 (0.0-0.03)	0.0 (0.0-0.0)	0.0 (0.0-0.05)	0.0 (0.0-0.0)	0.103

HIT: heparin-induced thrombocytopenia; ECMO: extracorporeal membrane oxygenation; p-value <0.001 concerning: [1]HIT suspicion vs. ECMO control, [2]HIT excluded vs. ECMO control.
