# Supplementary material for: Prevalence and outcomes of patients developing heparin-induced thrombocytopenia during extracorporeal membrane oxygenation
Source: PLoS One. 2022 Aug 8;17(8):e0272577. doi: 10.1371/journal.pone.0272577 (PMC9359525; doi:10.1371/journal.pone.0272577)
Supplement: S5 Fig — (PDF) [file pone.0272577.s011.pdf]

S5 Fig. Median antithrombin III on extracorporeal membrane oxygenation

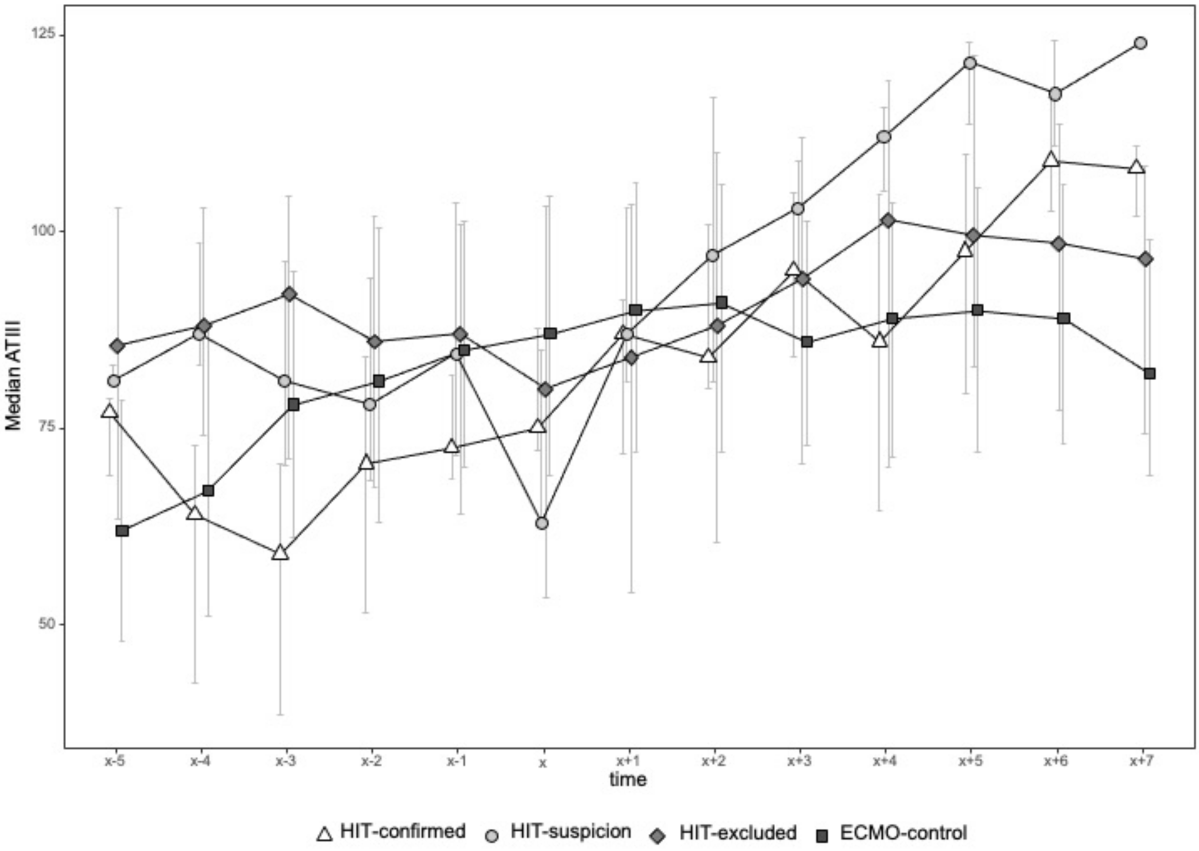

Trajectories of antithrombin III (ATIII in %) before and after suspicion of heparin-induced thrombocytopenia (HIT) according to the HIT-confirmed group, the HIT-suspicion group, the HIT-excluded group and the ECMO-control group. Data show median and interquartile range (q1-q3). Time axis in days from day x. x: day of HIT suspicion (change to alternative anticoagulation) or, for group ECMO-control day 7 of ECMO therapy (as median time to HIT on ECMO was 7,5 days). 35 patients were excluded because the ECMO was explanted within 3 days after changing of anticoagulation or they died within 3 days after changing of anticoagulation, to show the effect of the alternative anticoagulation on coagulation parameters. Occasional missings e.g. if ECMO duration was shorter than 7 days.
