# Supplementary material for: Prevalence and outcomes of patients developing heparin-induced thrombocytopenia during extracorporeal membrane oxygenation
Source: PLoS One. 2022 Aug 8;17(8):e0272577. doi: 10.1371/journal.pone.0272577 (PMC9359525; doi:10.1371/journal.pone.0272577)
Supplement: S8 Fig — (PDF) [file pone.0272577.s014.pdf]

S8 Fig. Median thrombocyte counts of veno-arterial vs. veno-venous extracorporeal membrane oxygenation of groups HIT-confirmed and HIT-suspicion

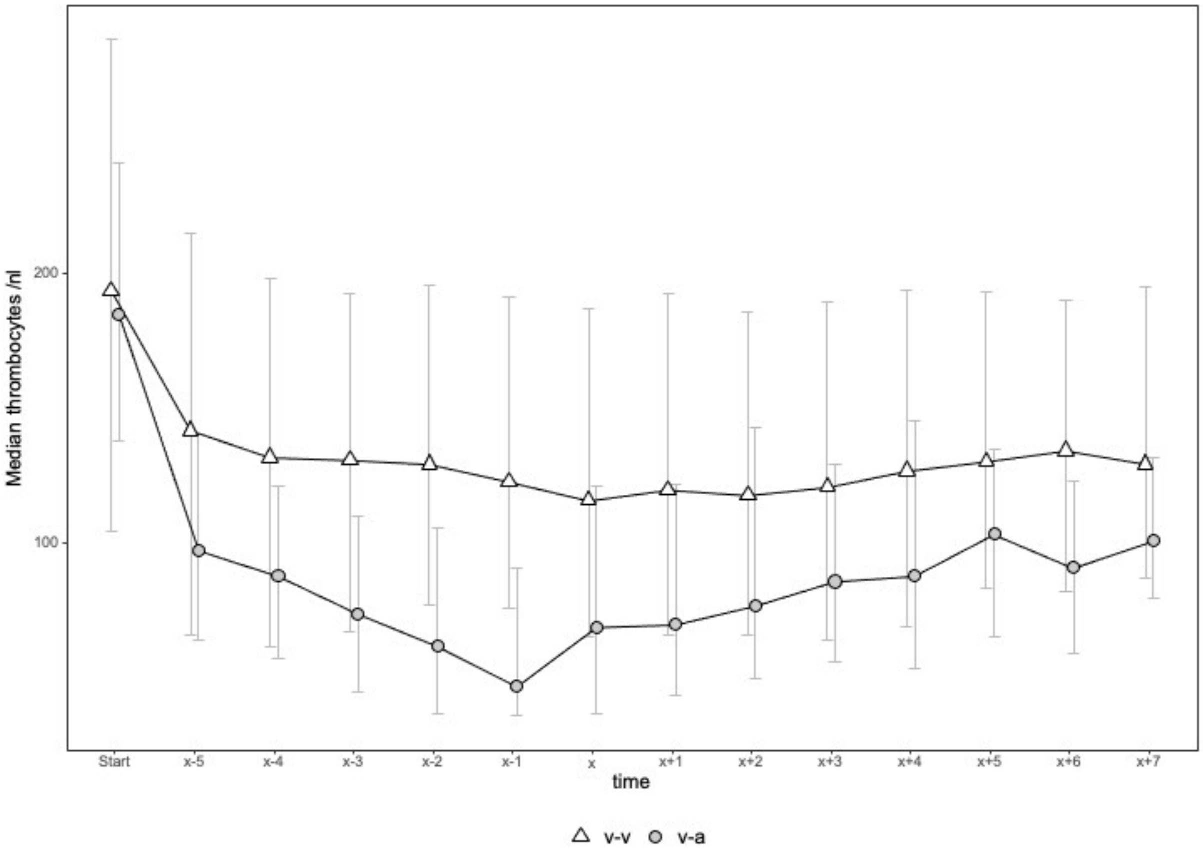

Trajectories of median platelet counts (/nl) before and after suspicion of heparin-induced thrombocytopenia (HIT) according to VV ECMO and VA ECMO of groups HIT-confirmed and HIT-suspicion. Data show median and interquartile range (q1-q3). Time axis in days from day x. x: day of HIT suspicion (change to alternative anticoagulation). Eleven patients were excluded in this figure because the ECMO was explanted within 3 days after changing of anticoagulation or they died within 3 days after changing of anticoagulation, to show the effect of the alternative anticoagulation on coagulation parameters. Occasional missings e.g. if ECMO duration was shorter than 7 days.
