# Supplementary material for: Prevalence and outcomes of patients developing heparin-induced thrombocytopenia during extracorporeal membrane oxygenation
Source: PLoS One. 2022 Aug 8;17(8):e0272577. doi: 10.1371/journal.pone.0272577 (PMC9359525; doi:10.1371/journal.pone.0272577)
Supplement: S9 Fig — (PDF) [file pone.0272577.s015.pdf]

S9 Fig. Thrombocyte counts and day of circuit exchange in individual patients of group HIT-confirmed

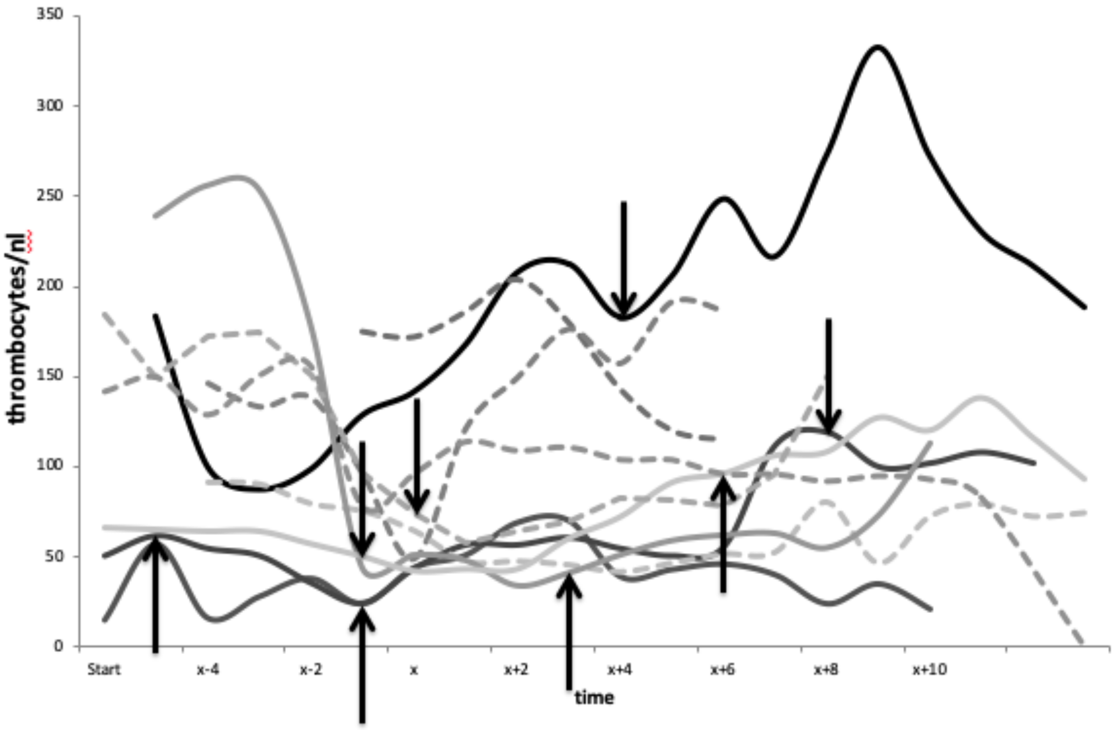

Trajectories of platelet counts of individual patients with confirmed HIT before and after suspicion of heparin-induced thrombocytopenia (HIT) and impact of circuit exchange (arrow). Time axis in days from day x. x: day of HIT suspicion (change to alternative anticoagulation). Six patients of group HIT-confirmed were excluded in this figure because the ECMO was explanted within 3 days after changing of anticoagulation or they died within 3 days after changing of anticoagulation, to show the effect of the alternative anticoagulation on coagulation parameters.
