# Supplementary material for: Prevalence and outcomes of patients developing heparin-induced thrombocytopenia during extracorporeal membrane oxygenation
Source: PLoS One. 2022 Aug 8;17(8):e0272577. doi: 10.1371/journal.pone.0272577 (PMC9359525; doi:10.1371/journal.pone.0272577)
Supplement: S10 Fig — (PDF) [file pone.0272577.s016.pdf]

S10 Fig. Thrombocyte counts and day of circuit exchange in individual patients of group HIT-suspicion

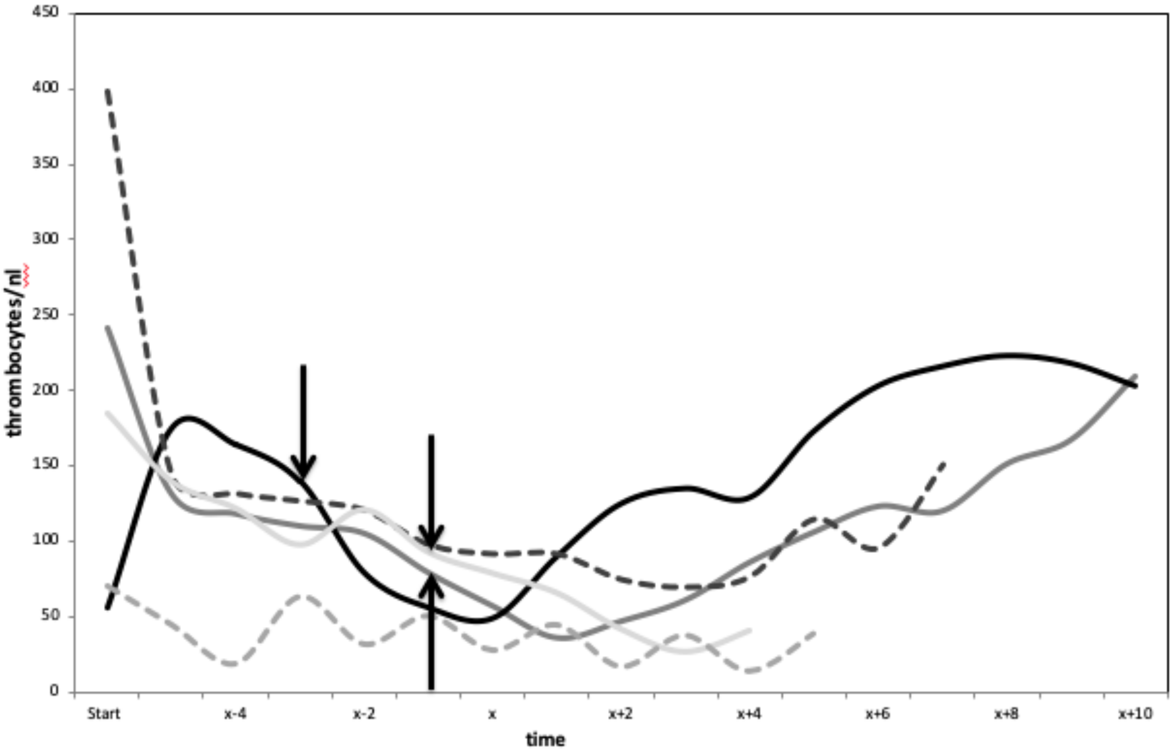

Trajectories of platelet counts of individual patients of group HIT-suspicion before and after suspicion of heparin-induced thrombocytopenia (HIT) and impact of circuit exchange (arrow). Time axis in days from day x. x: day of HIT suspicion (change to alternative anticoagulation). 5 patients of group HIT-suspicion were excluded in this figure because the ECMO was explanted within 3 days after changing of anticoagulation or they died within 3 days after changing of anticoagulation, to show the effect of the alternative anticoagulation on coagulation parameters.
