# Supplementary material for: Prevalence and outcomes of patients developing heparin-induced thrombocytopenia during extracorporeal membrane oxygenation
Source: PLoS One. 2022 Aug 8;17(8):e0272577. doi: 10.1371/journal.pone.0272577 (PMC9359525; doi:10.1371/journal.pone.0272577)
Supplement: S11 Fig — (PDF) [file pone.0272577.s017.pdf]

S11 Fig. Comparison of heparin-coated ECMO-systems vs. non-heparin-coated ECMO-systems in relation to thrombocyte counts of individual patients in the group HIT-confirmed

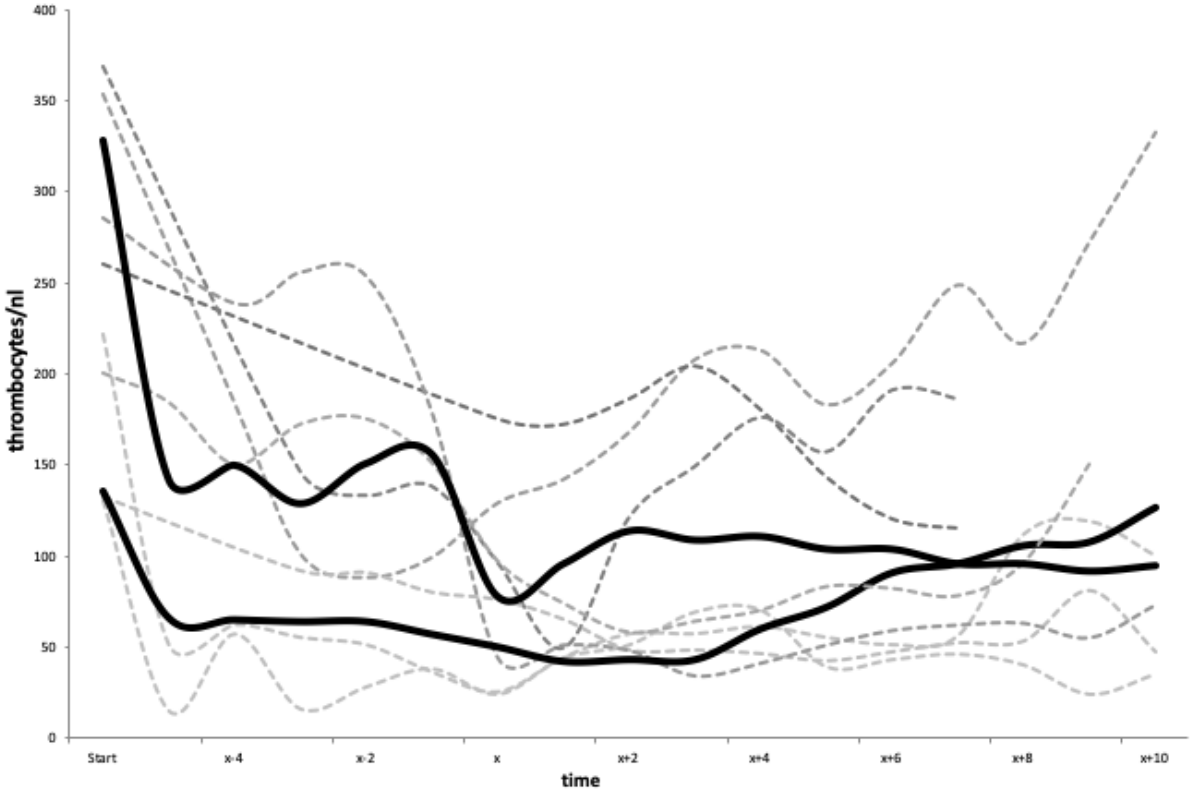

Trajectories of platelet counts of individual patients with confirmed HIT before and after suspicion of heparin-induced thrombocytopenia (HIT) of heparin-coated vs. non-heparin-coated ECMO-systems. Time axis in days from day x. x: day of HIT suspicion (change to alternative anticoagulation); black line = non-heparin-coated ECMO-systems (Phosphorylcholin coating, Sorin® / Livanova®); dashed line = heparin-coated ECMO-systems (Bioline coating Maquet® / Getinge® and Xenios® / Fresenius Medical Care®). Six patients were excluded because the ECMO was explanted within 3 days after changing of anticoagulation or they died within 3 days after changing of anticoagulation, to show the effect of the alternative anticoagulation on coagulation parameters.
